# Timescales modulate optimal lysis–lysogeny decision switches and near-term phage reproduction

**DOI:** 10.1093/ve/veac037

**Published:** 2022-05-23

**Authors:** Shashwat Shivam, Guanlin Li, Adriana Lucia-Sanz, Joshua S Weitz

**Affiliations:** School of Physics, Georgia Institute of Technology, Atlanta, GA, USA; Interdisciplinary Graduate Program in Quantitative Biosciences, Georgia Institute of Technology, Atlanta, GA, USA; School of Biological Sciences, Georgia Institute of Technology, Atlanta, GA, USA; School of Biological Sciences, Georgia Institute of Technology, Atlanta, GA, USA; School of Physics, Georgia Institute of Technology, Atlanta, GA, USA; Institut d’Biologie, École Normale Supérieure, Paris, France; School of Electrical and Computer Engineering, Georgia Institute of Technology, Atlanta, GA, USA

**Keywords:** viral ecology, evolution, mathematical modeling, control theory

## Abstract

Temperate phage can initiate lysis or lysogeny after infecting a bacterial host. The genetic switch between lysis and lysogeny is mediated by phage regulatory genes as well as host and environmental factors. Recently, a new class of decision switches was identified in phage of the SPbeta group, mediated by the extracellular release of small, phage-encoded peptides termed arbitrium. Arbitrium peptides can be taken up by bacteria prior to infection, modulating the decision switch in the event of a subsequent phage infection. Increasing the concentration of arbitrium increases the chance that a phage infection will lead to lysogeny, rather than lysis. Although prior work has centered on the molecular mechanisms of arbitrium-induced switching, here we focus on how selective pressures impact the benefits of plasticity in switching responses. In this work, we examine the possible advantages of near-term adaptation of communication-based decision switches used by the SPbeta-like group. We combine a nonlinear population model with a control-theoretic approach to evaluate the relationship between a putative phage reaction norm (i.e. the probability of lysogeny as a function of arbitrium) and the extent of phage reproduction at a near-term time horizon. We measure phage reproduction in terms of a cellular-level metric previously shown to enable comparisons of near-term phage fitness across a continuum from lysis to latency. We show the adaptive potential of communication-based lysis–lysogeny responses and find that optimal switching between lysis and lysogeny increases the near-term phage reproduction compared to fixed responses, further supporting both molecular- and model-based analyses of the putative benefits of this class of decision switches. We further find that plastic responses are robust to the inclusion of cellular-level stochasticity, variation in life history traits, and variation in resource availability. These findings provide further support to explore the long-term evolution of plastic decision systems mediated by extracellular decision-signaling molecules and the feedback between phage reaction norms and ecological context.

## 1. Introduction

Temperate phages like P1, P2 (Bertani 2004), phage *λ* (Kourilsky 1973), and phage *µ* (Howe 1998) can initiate lysis or lysogeny after infection, whereas virulent phages can only initiate lysis. In a lytic pathway, the virus hijacks host cellular machinery to produce viral RNA and proteins, replicate the viral genome, assemble mature virus particles, lyse (and kill) the host cell, and ultimately release virus particles (Young 1992) into the extracellular environment. In a lysogenic pathway the viral genome is integrated into the host genome. The stably integrated viral genome, i.e. the ‘prophage’, is then replicated along with the host (Lwoff 1953) where the prophage is vertically transmitted to progeny cells. The integration of the phage genome often confers benefits to the lysogen, including immunity to infection and direct benefits to growth (Abedon 2015; Bondy-Denomy *et al.* 2016; Harrison and Brockhurst 2017; Correa *et al.* 2021). Finally, the prophage can be induced, leading to the excision of the viral genome and the re-initiation of the lytic pathway, including the release of virus particles (Oppenheim *et al.* 2005).

Despite the existence of multiple pathways for propagation of viral genomes, the evolutionary ‘success’ of a phage has often been measured in terms of the quantity of viral particles generated via lysis or in terms of phage abundance in absolute terms (Cobián Güeme *et al.* 2016). However, viral replication need not include production of virus particles, at least not in the short term. Instead, measuring phage life cycles in terms of infected cells allows a comparison between strategies that lie on a continuum between lysis and lysogeny (Weitz *et al.* 2019; Li *et al.* 2020; Correa *et al.* 2021). In cell-centric terms, the near-term Malthusian fitness is defined as the growth rate of phage-infected cells, irrespective of whether these cells are part of the lysogenic or lytic pathways. Lysis is favored relative to lysogeny in environments with higher susceptible cell density and longer extracellular survival of viral particles. As such, the near-term Malthusian fitness depends not only on phage strategies but on the ecological context.

In prior work using this cell-centric metric (Stewart and Levin 1984; Berngruber *et al.* 2013; Wahl 2018; Weitz *et al.* 2019; Li *et al.* 2020), host abundances were shown to influence the invasibility of phage. Increasing susceptible host densities increases the chance that phage particles released via lysis encounter and infect new hosts. In contrast, given low susceptible densities, phage that integrates into hosts can proliferate as lysogens while competing with fewer cells. These findings, framed in terms of the invasion fitness criteria for temperate phage, suggest that lysogeny can be an adaptive benefit in the near term, particularly when host cell densities are low. Extrapolating from these prior findings, an intracellular sensing mechanism that allows phage to adjust life cycle decisions after infection given variation in host cellular density could be of adaptive benefit.

The study of the switch between lysis and lysogeny remains an important field of study, given the prevalence of prophages in bacterial chromosomes and their impact on bacterial behavior, pathogenicity, and evolution (Lwoff 1953; Ptashne 2004; Abedon 2015; Bondy-Denomy *et al.* 2016; Harrison and Brockhurst 2017; Correa *et al.* 2021). Seminal work showed that the population-level frequency of lysogeny in phage *λ* increased with increasing ratios of phage to hosts (Kourilsky 1973). Hence, when phage were relatively more abundant than bacterial hosts, phage infections tended to lead to lysogeny, rather than lysis. Recent work using single-cell imaging methods revealed that the probability of lysogeny increases with increasing cellular multiplicity of infection (from $\sim 20$ per cent given a single phage to $\sim 80$ per cent given five coinfecting phages) (Zeng *et al.* 2010; Golding *et al.* 2019). These shifts in cellular fate reflect a combination of interactions between phage-specific gene regulatory circuits and the ecological context (which drives changes in the multiplicity of infection) (Weitz *et al.* 2008; Joh and Weitz 2011; Yao *et al.* 2021).

Lysis–lysogeny decisions are also mediated by small signaling molecules released to the environment. For example, the phage *VP882* uses the quorum sensing molecule acyl-homoserine lactone (Als) produced by the host, *Vibrio cholerae*, to activate genes responsible for lysis (Silpe and Bassler 2018). In this case, an increase in host density translates to an increase in Als levels in the medium, and the probability of lysogeny decreases. Likewise, phages of the SPbeta group utilize the quorum sensing molecule arbitrium for lysis–lysogeny decisions (Erez *et al.* 2017). Upon infection, the phage genetic arbitrium system regulates the release of a small peptide from infected cells that increase the probability that future infections are lysogenic, rather than lytic. In the absence of arbitrium in the environment, a receptor activates *aimX* expression, which in turn directs the phage to a lytic cycle. In the presence of arbitrium, the receptor binds to the peptide and the expression of *aimX* is repressed, which preferentially initiates the lysogenic cycle. A recent modeling study (Doekes, Mulder and Hermsen 2021) deduced that the communicating phage outcompetes non-communicating phage, particularly when host densities become low. The selection for lysis when susceptible host cells are abundant and for lysogeny when susceptible cells become scarce is hypothesized to increase the long-term growth rate of phage that utilizes a communicating strategy. This previous modeling study assumed the presence of a rapid shift in response. Arbitrium systems may also include additional quorum sensing circuits that increase the efficiency of switching between lysis and lysogenic pathways (Bernard, Lopez and Bapteste 2021). For example, phage phi3T employs two communication systems, arbitrium for lysis–lysogeny and Rap*φ*-Phr*φ* that downregulates host defenses and confers a fitness advantage to lysogenized cells. These examples demonstrate a connection between the cellular fate of infection and the inter-host exchange of small molecules. In all cases, the levels of fate-influencing molecules (Als or arbitrium) are connected to the population context. However, it is not well understood which strategy would be optimal over what timescales, given the variation in environmental conditions.

A long-standing rationale for the adaptive benefit of lysogeny is that integration by phage into their bacterial hosts enables viruses to persist even when fluctuations drive bacterial densities to low levels. Using a nonlinear model of population dynamics between phage and bacteria, Stewart and Levin (Stewart and Levin 1984) found that temperate phage densities were higher than that of virulent phage densities following transient periods of poor conditions for bacterial survival. In the context of a widespread lytic infection, as the susceptible population reduces, the benefit of lysis diminishes. Thus, in the early stages of infection, higher virulence may be selected and as bacterial populations decrease, more temperate phages are successful (Berngruber *et al.* 2013), allowing the bacterial population to recover. A putative inverse relation between susceptible population density and the benefit of lysogenic pathways has been suggested using evolutionary models (Wahl 2018) as well as conceptual models of stochastic growth (Avlund *et al.* 2009; Maslov and Sneppen 2015). By extension, if an infecting phage could estimate bacterial cell densities or the fraction of infected cells, then there is a potential adaptive benefit for the evolution of a decision switch that could modulate the rate of initiating lysis or lysogeny.

Here, we focus on phage of the SPbeta group and the arbitrium system to explore the potential near-term adaptive benefits of arbitrium-dependent decision switches. In doing so, we utilize a control-theoretic framework embedded as part of a nonlinear population model to identify optimal responses of phage to variation in arbitrium concentration. We select a time horizon to maximize the near-term phage reproduction, as measured in terms of the increase in newly infected cells and lysogens—which correspond to epidemiological birth states for phage. This allows us to explore the link between a continuum of strategies, given the variation in near-term selection horizons. As we show, rapidly switching from lysis to lysogeny with increasing arbitrium represents a control-theoretic optimum to maximize cell-centric measures of phage reproduction in the near term. In the Discussion we consider ways to extend the formalism to evaluate the long-term adaptation and speculate on how the evolution of communication-based systems can help shape long-term phage strategies and fitness.

## 2. Methods

### 2.1 Population dynamics model of virus–bacteria–arbitrium system

**Figure 1.**
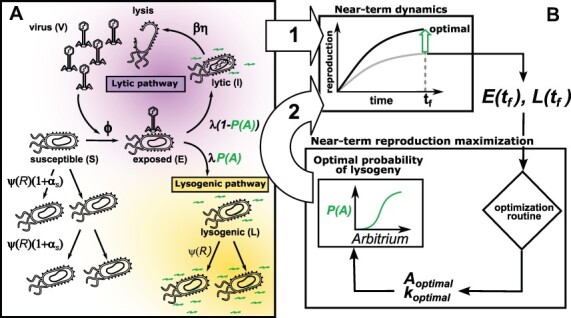
Schematic of the model. (A) We consider a batch culture of susceptible bacteria, *S*, infected by free temperate phage, *V*, given limited resources, *R*. Early in the infection, exposed bacteria, *E*, sense the concentration of arbitrium quorum sensing molecule, *A* (in green), from the environment, and a decision between lysis and lysogeny is made based on the optimal probability function *P*(*A*). Lytic infections (in purple), *I*, result in the destruction of the infected bacteria and the release of a burst of new phage to the environment. Lysogenic infections (in yellow) result in the internalization of the phage in the bacterial chromosome, and the resulting lysogen, *L*, continues its replication cycle. Both infection pathways produce and release arbitrium molecules into the environment. (B.1) We use final population sizes of infected cells (*L* + *E*) at a given time horizon, *t*_*f*_, to (B.2) optimize the function of probability of lysogeny, by finding the optimal arbitrium switching point, *A*_0_, and shaping parameter, *k*, that maximizes cell-centric metric of phage reproduction at the given *t*_*f*_.

We consider the population dynamics of temperate phage in a resource-explicit model that includes an explicit representation of infections including cells that are either susceptible (*S*), exposed (*E*), lytic-fated infected (*I*), or lysogens (*L*), as well as virus particles (*V*), see Fig. 1. The exposed cells have been infected but denote the situation in which the virus has not yet committed to either the lytic pathway (turning it into an lytic-fated infected, *I*, cell) or the lysogenic pathway (turning it into a lysogen, *L*). The lysis–lysogeny decisions are guided by the concentration of a small quorum sensing peptide *A* (i.e. arbitrium) (Erez *et al.* 2017). For the most general case, we assume that arbitrium molecules are produced from lytic-fated infected cells and lysogens at some (possibly different) generation rate. Here, we denote the probability of lysogeny as a response function of *A*, i.e. *P*(*A*); consequently, a single exposed cell *E* has a probability $1 - P(A)$ when entering the *I* state. We represent this model in terms of systems of nonlinear ordinary differential equations, which can be written as follows

(2.1) $$ \begin{aligned} \dot{R}&= \overbrace{J}^{\text{influx}} - \overbrace{e\psi(R)(L+(1+\alpha _S)S)}^{\text{nutrient consumption}} - \overbrace{d_RR}^{\text{decay}}\\ \dot{S}& = \overbrace{\psi(R)(1+\alpha _S)S}^{\text{growth}} - \overbrace{\phi SV}^{\text{infection}} - \overbrace{d_SS}^{\text{decay}}\\ \dot{E}& = \overbrace{\phi SV }^{\text{infection}} - \overbrace{\lambda E}^{\text{transition}} - \overbrace{d_EE}^{\text{decay}}\\ \dot{L}& = \overbrace{\psi(R)L}^{\text{growth}} + \overbrace{P(A)\lambda E }^{\text{lysogenic infection}} - \overbrace{d_LL}^{\text{decay}}\\ \dot{I}& = \overbrace{(1-P(A))\lambda E}^{\text{lytic infection}} - \overbrace{\eta I}^{\text{lysis}} - \overbrace{d_II}^{\text{decay}}\\ \dot{V}& = \overbrace{\beta \eta I}^{\text{burst}} - \overbrace{\phi NV }^{\text{infection}} - \overbrace{d_VV}^{\text{decay}}\\ \dot{A}& = \overbrace{k_LL+ k_II}^{\text{production}} - \overbrace{d_AA}^{\text{decay}}, \end{aligned} $$

where *S*, *E*, *L*, *I*, *V*, and *A* denote the densities of susceptible cells, exposed infected cells, lysogens, lytic-fated infected cells, virus particles, and arbitrium molecules, respectively. $N = S+E+L+I$ is the cell density of all bacterial cells. Only viruses interacting with a susceptible cell lead to a new infection. We utilize a multistage infected cell model to ensure that latent periods are finite and of average duration in the event of transition to lysis: 1/*λ* + 1/*η* (see Levin, Stewart and Chao 1977; Weitz 2015; Mitarai, Brown and Sneppen 2016) for more details on latent period distributions). Given the resource-explicit dynamics, *ψ(R)* = *μ_max_(R/(R + R_in_*)) is the Monod equation, where *µ*_*max*_ and *R*_*in*_ are the maximal cellular growth rate and the half-saturation constant, *J* and *d*_*R*_ are the influx and decay rates of resources, *e* is the conversion efficiency, and *α*_*S*_ is the selection coefficient that measures the relative difference in the reproductive output between lysogens and susceptible cells. Parameter *ϕ* is the adsorption rate and $d_S, d_E, d_L$, and *d*_*I*_ are the cellular death rates of susceptible cells, exposed infected cells, lysogens, and lytic-fated infected cells, respectively. In addition, *d*_*V*_ and *d*_*A*_ are the decay rate of viruses and molecules. *λ* is the transition rate from exposed cells to the fate-determined cells, *η* is the lysis rate, and *β* is the burst size. The arbitrium molecules are produced by infected cells: *L* and *I* at a production rate of *k*_*L*_ and *k*_*I*_, respectively. According to Erez *et al.* (2017), the binding of the arbitrium molecule and the *AimR* receptor of phage phi3T saturates around 500 nM ($3\times 10^{14}$ molecules/ml), which we set as the target accumulation level of arbitrium after 24 h. These target accumulation levels are reached in our simulations when we set $k_{L,I} = 5 \times 10^7$ molecules/cell h^‒1^ ($\sim 2 \times 10^6$ molecules produced per bacterium), which fall within physiological levels of bacterial protein production (Milo 2013). For simplicity, we assume that lysogens and lytic-fated infected cells have identical arbitrium production rates. The model does not account for cellular active transport mechanisms, which could imply that lower external concentrations of arbitrium (and lower production rates) could nonetheless generate physiologically relevant internal concentrations of arbitrium. Spontaneous induction within a physiological range (10^−5^ to $10^{-2}\ h^{-1} $) has a negligible effect on arbitrium responsiveness even for the longer time horizons considered in this work (less than 2 per cent difference in arbitrium switching concentrations). This is an expected outcome, as we focus on short-term fitness. Therefore, spontaneous induction is ignored in the model (see Discussion for potential ways to extend the current model to include the induction process when assessing long-term fitness for the communicating phage). All parameter values used are listed in Table A1.

For tractability, we assume that the probability function $P_{\theta}: \mathbb{R}^+ \rightarrow [0,1]$ lies in the class of sigmoid functions parameterized by *θ*, where $\theta = [k ,A_{0}]^{T}$, and contains shaping parameter, $k \in \mathbb{R}$, and the switching point, $A_0 \in \mathbb{R}^{+}$. In doing so, the probability of lysogeny has the form:

(2.2) $$ P_{\theta}(A) = \frac{1}{1+e^{-k\left(\frac{A}{A_0} - 1\right)}}, $$

which is the arbitrium molecule sensing function. Both *k* and *A*_0_ are optimized by the optimization framework (Fig. 1b) in order to maximize fitness at a given time horizon, *t*_*f*_ (see Section 2.4).

### 2.2 Near-term reproduction and growth rate

We quantify viral reproduction in terms of infected cells in order to compare pure lytic and context-dependent lysis–lysogenic strategies. We use a standard definition of Malthusian fitness, expressed as the time-normalized growth rate of phage-infected cells, from either the lytic or lysogenic pathway (e.g. Day and Otto (2001)). Newly infected cells can only be produced by the epidemiological birth states, i.e. the lysogens and exposed cells (Weitz *et al.* 2019; Li *et al.* 2020). Let the time horizon for optimization be denoted as *t*_*f*_. The time-normalized growth rate *ρ* denotes the effective reproductive rate of these infected cells and is given as:

$$ \rho(t_{f}) = \frac{1}{t_{f}} \text{log} \left[\frac{L(t_f) + E(t_f)}{\bar{E}_{0}} \right]\ ,\quad \bar{E}_{0} = \frac{\phi S_0}{\phi S_0 + d_V}V_0, $$

where $\bar{E}_{0}$ is the average number of cells infected in the first generation. The first factor of $\bar{E}_{0}$ denotes the probability that a free virus particle is adsorbed into a susceptible cell before it decays. This measure of phage reproduction assumes that the survival of temperate phages using a communication system is contingent on the growth of lysogens and exposed hosts potentially entering lysogeny at the final time. For example, if phage particles cannot survive to a new environment, then lysogens will represent the predominant route for long-term phage fitness. As such, we also consider the optimal decision switch when measuring phage reproduction in terms of lysogen production at a fixed time horizon (see Appendix for related results).

### 2.3 Optimization framework for reproduction maximization

We formulate an optimization problem over different probability functions with the aim of achieving a maximum of near-term phage reproduction as defined in the previous section using a cell-centric metric. By controlling the lysis–lysogeny decisions $p_{\theta}(A)$ (more precisely, the variable $\theta = [k ,A_{0}]^{T}$), the objective is to maximize the epidemiological birth states at the final time. In order to do so, we define a payoff as a function of *θ*, $Q(\theta) = L(t_f) + E(t_f)$. Given the initial conditions, the system dynamics are dependent on the probability function determining the switch from lysis to lysogeny. A change in *θ* affects the probability function and changes the exposed cells and lysogens produced at the final time. Thus, the optimization problem can be written as

(2.3) $$ \max_{\theta \in \Omega} Q(\theta) = \max_{\theta \in \Omega} L(t_f) + E(t_f), $$

where $\Omega = \mathbb{R} \times \mathbb{R}^{+}$.

We implement a gradient ascent algorithm to numerically compute the optimal *θ* for optimization problem (2.3). In order to do so, we need to evaluate the gradient of *Q* with respect to *θ*. Let the state vector of the system be represented by $X \in \mathbb{R}^{n}$, (here *n* = 7) and let the dependence of the payoff function on the final state be given by Ψ, i.e. $Q = \Psi(X(t_f)) = (e_{3} + e_{4})^{T}X(t_{f})$ as *Q* is linear. Here *e*_*i*_ represents the *i*^th^ unit vector. We represent the nonlinear system (2.1) as

$$ \dot{X}=f(X,\theta),\ \ X(0) = X_0, $$

where $f(X, \theta) \in \mathbb{R}^{n}$, is the right-hand side (RHS) of system (2.1).

Note that *Q* is a function of the final state, $X(t_f)$, where $X(t_f)$ is a function of *θ*, thus we apply chain rule for finding the required derivative. In doing so, the derivative of *Q* with respect to *θ* takes the form of

(2.4) $$ \frac{dQ}{d\theta} = \frac{\partial \Psi}{\partial X}(X(t_f))\frac{dX(t_f)}{d\theta}, $$

where $\frac{\partial \Psi}{\partial X}(X(t_f)) = [0, 0, 1, 1, 0, 0, 0]$ is a row vector. There is no closed-form algebraic expression for the terminal state derivative, i.e. $\frac{dX(t_f)}{d\theta}$. To evaluate it, we let $\xi(t) = \frac{dX(t)}{d\theta}$, and using $\frac{d}{dt}\frac{dX(t)}{d\theta} = \frac{d}{d\theta}\frac{dX(t)}{dt}$, we have

(2.5) $$ \begin{aligned} \dot{\xi}(t) &= \frac{d}{d\theta}\frac{dX(t)}{dt}\\ &= \frac{d}{d\theta}f(X(t), \theta)\\ &= \frac{\partial f(X(t), \theta)}{\partial X}\xi + \frac{\partial f(X(t), \theta)}{\partial \theta}. \end{aligned} $$

Note that system  (2.5) is a linear system. Let the state transition matrix for (2.5) be Φ. Then, we obtain

$$ \xi(t) = \int_{t_0}^{t}\Phi(t,\tau)\frac{\partial f}{\partial \theta}(X(\tau), \theta)d\tau. $$

Substituting the terminal state derivative $\xi(t_f)$ into Eq.  (2.4), the derivative of *Q* can be written as

(2.6) $$ \begin{aligned} \frac{dQ}{d\theta} &= \frac{\partial \Psi}{\partial X}(X(t_f))\int_{t_0}^{t_f}\Phi(t_f,\tau)\frac{\partial f}{\partial\theta}(X(\tau), \theta)d\tau\\ &= \int_{t_0}^{t_f}\frac{\partial \Psi}{\partial X}(X(t_f))\Phi(t_f,\tau)\frac{\partial f}{\partial\theta}(X(\tau), \theta)d\tau. \end{aligned} $$

Here, we define the *costate* as $\pi^T(\tau) = \frac{\partial \Psi}{\partial X}(X(t_f))\Phi(t_f,\tau)$, with $\pi^T(t_f) = \frac{\partial \Psi}{\partial X}(X(t_f)) $. Differentiating the costate, we have

$$ \dot{\pi}^T(\tau) = -\pi^T \frac{\partial f}{\partial X}(X(\tau, \theta)). $$

The costate trajectory $\pi(t)$ can be solved numerically in backward direction using the terminal boundary condition. Then, we substitute the costate in Eq.  (2.6) and obtain

$$ \frac{dQ}{d\theta} = \int_{t_0}^{t_f}\pi^T(\tau)\frac{\partial f}{\partial \theta}(X(\tau, \theta)) d\tau. $$

Since the costate trajectory can be computed by numerical integration of the costate differential equation, the derivative of *Q* with respect to *θ* can be computed.

The gradient descent algorithm does not guarantee the convergence to the global maxima. To circumvent the issue of convergence to a local maxima, we perform ‘naive sampling’ before the gradient ascent search. First, we divide the search space into grids and find the payoff function’s value at the center of each grid. Then, we select the center point which gives the largest value for *Q* and choose that as the starting point of the gradient descent algorithm. If the grids are small enough, this naive sampling approach will be equivalent to a ‘brute-force search’ of the complete parameter space. Here, we employ Armijo’s step size in gradient ascent (Armijo 1966). This process is iterated until the increase in cost function between iterations is lower than the preset termination value. Once the optimal parameters are reached, they can be used to obtain the optimal probability function.

### 2.4 Arbitrium sensing mechanism and expected lysogeny probability

The inclusion of imperfect sensing is modeled via a sigmoidal response function that is dependent on the estimated concentration instead of the true concentration. Let the concentration of arbitrium molecule in the medium be *A*  $\text{molecules/ml}$. We assume a typical bacterial size of $\sim 1\ \mu m^3$. As such, the number of molecules per equivalent cell volume would be on the order of $A/10^{12}$ (assuming nearly uniform concentration in the medium), and the estimated value will be denoted as $\widehat{A}$.

Assume there are *E* exposed cells, numbered as $\{E_i\}^E_{i=1}$. Let the true concentration of arbitrium molecule in the medium be *A* and let the sensed concentration be $\{\widehat{A}_i\}^E_{i=1}$ for each phage in an exposed cell. For convenience, we define $\sigma_{a, b}(x) = 1/(1 + e^{-a(\frac{x}{b} - 1)})$ as a standard sigmoid function parameterized by *a* and *b*. In doing so, we can write the probability of lysogeny $p_{\theta}(A) = \sigma_{k, A_{0}}(A)$. The probability of lysogeny for exposed cell *E*_*i*_ can be written as

(2.8) $$ P\left(E_i\xrightarrow{}L_i|\widehat{A}_i\right) = \sigma_{k, A_{0}}(\widehat{A}_{i}) $$

as it only depends on the sensed concentration. Note that *A*_0_ is the switching point for the sigmoid and *k* is a shaping parameter, which are the two parameters to be optimized.

For each individual cell with a sensed concentration of $\widehat{A}_i$, we denote its initiation of lysogeny with an indicator function, ${1}_{\{E_i\xrightarrow{}L_i\}}$, a binary random variable. Then, the total number of exposed cells that undergo lysogeny, denoted by *L*_new_, can be written as

$$ L_{\text{new}} = \sum_{i=1}^E {1}_{\{E_i\xrightarrow{}L_i\}}. $$

The conditional expectation of *L*_new_ given the estimated concentrations $\{\widehat{A}_i\}^E_{i=1}$ is

(2.9) $$ \mathbb{E}[L_{\text{new}}|\{\hat{A}_i\}^E_{i=1}] = \sum_{i=1}^E \sigma_{k, A_{0}}(\widehat{A}_{i}). $$

Assuming $\widehat{A}_1, \widehat{A}_2, {\ldots}, \widehat{A}_E$ are independent and identically distributed Poisson random variables, using the iterated expectation law on (2.9), we have

$$\eqalign{ \mathbb{E}[L_{\text{new}}] &= \mathbb{E}_{\{\widehat{A}_i\}^E_{i=1}}\left[\mathbb{E}\left[L_{\text{new}}|\{\hat{A}_i\}^E_{i=1}]\right]\right]\\ &=\mathbb{E}_{\{\widehat{A}_i\}^E_{i=1}}\left[\sum_{i=1}^E \sigma_{k, A_{0}}(\widehat{A}_{i})\right]\\ &= \sum_{i = 1}^{E} \mathbb{E}_{\widehat{A}_i \sim \text{Poisson}(A)}\left[\sigma_{k, A_{0}}(\widehat{A}_{i})\right]\\ &= \left(\mathbb{E}_{\widehat{A}_i \sim \text{Poisson}(A)}\left[\sigma_{k, A_{0}}(\widehat{A}_{i})\right]\right) E.} $$

We approximate the first moment of the lysis–lysogeny decision function of the estimated concentration, i.e. $\mathbb{E}_{\widehat{A}_i \sim \text{Poisson}(A)}\left[\sigma_{k, A_{0}}(\widehat{A}_{i})\right]$, using Taylor expansions provided that $\sigma_{k, A_{0}}$ is sufficiently smooth (twice continuously-differentiable at least) and the moments of $\widehat{A}_{i}$ are finite. Taking the local expansion of $\sigma_{k, A_{0}}(\widehat{A}_{i})$ around *A* yields

$$\eqalign{ \mathbb{E}_{\widehat{A}_i \sim \text{Poisson}(A)}\left[\sigma_{k, A_{0}}(\widehat{A}_{i})\right] &= \mathbb{E}_{\widehat{A}_i \sim \text{Poisson}(A)}\left[\sigma_{k, A_{0}}(A + (\widehat{A}_{i} - A))\right]\\ &\approx \mathbb{E}_{\widehat{A}_i \sim \text{Poisson}(A)}\left[\sigma_{k, A_{0}}(A) + \sigma_{k, A_{0}}^{\prime}(A)(\widehat{A}_{i} - A)\right.\nonumber\\ &\qquad\qquad\qquad\qquad+ \left.\frac{1}{2}\sigma_{k, A_{0}}^{\prime\prime}(A)(\widehat{A}_{i} - A)^{2}\right]\\ &= \sigma_{k, A_{0}}(A) + \frac{1}{2}\sigma_{k, A_{0}}^{\prime\prime}(A)\text{Var}[\widehat{A}_{i}]\\ &= \sigma_{k, A_{0}}(A) + \frac{A}{2}\sigma_{k, A_{0}}^{\prime\prime}(A),} $$

where the first term corresponds to the mean lysis–lysogeny response of estimated concentration and the second term corresponds to the *propagation of uncertainty*. Hence, we approximate the expectation by the first three terms from the Taylor series. Then, we can compute the expected number of exposed cells that undergo lysogeny as

(2.10) $$ \mathbb{E}[L_{\text{new}}] \approx \big[ \sigma_{k, A_{0}}(A) + \frac{A}{2}\sigma_{k, A_{0}}^{\prime\prime}(A)\big]E. $$

The subsequent results in all sections account for sensing noise and use (2.10) for estimating the lysogeny probability.

### 2.5 Simulation Details

The parameter values used for these results are given in Table A1. The initial concentrations of susceptible and viral particles are 10^7^ cells/ml and 10^5^ phage/ml, respectively, (Multiplicity of infection, MOI = 0.01) and the rest are set to 0. The initial resource concentration is 40 *µ* g/ml with no influx, except when the effect of resource level on switching point is studied (where the values used are explicitly stated in text). Simulation code is written in MATLAB R2020a and is available for download at https://github.com/WeitzGroup/ArbitriumLysisLysogeny.

## 3. Results

### 3.1 Analysis of fixed probability decision strategies

**Figure 2.**
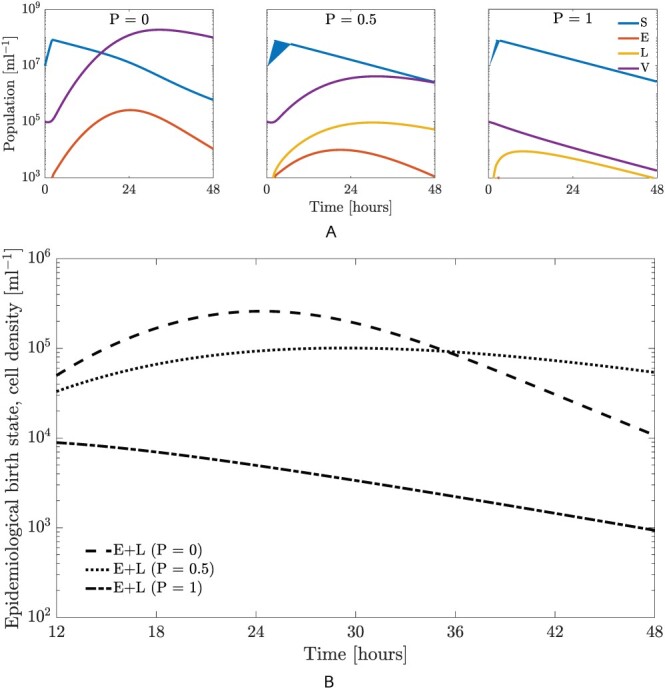
Temperate phage–bacterial infection dynamics for different fixed probabilities of lysogeny (*P* = 0, *P* = 0.5, or *P* = 1 where *P* is the probability of lysogeny) for 48 h with an MOI of 0.01. The model parameters are given in Table A1. (A) Population dynamics for the system when a fixed strategy is employed over a period of 48 h. The three panels correspond to an obligately lytic (*P* = 0, left panel), stochastic (*P* = 0.5, middle panel), and purely lysogenic (*P* = 1, right panel) strategy, respectively. (B) Comparison of total birth states, i.e. aggregate population of lysogens and exposed cells, for the three fixed strategies. The optimal fixed strategies with highest birth states population vary with time. Specifically, obligately lytic strategy is favored for relative short timescales (from 12 to 36 h). Beyond this, the mixed strategy (*P* = 0.5) is favored.

We begin by evaluating the population dynamics of host–phage systems in which phage has a fixed probability to initiate lysogeny, *P*. We first consider three cases—that of purely lytic (*P* = 0), mixed (*P* = 0.5), and purely lysogenic (*P* = 1) phage (first, second, and third panels of Figure 2). The system is initialized with a low-resource density, an MOI of 0.01, where MOI is the initial virus–host ratio, and then simulated for 48 h.

The first, second, and third panels of Figure 2 show the dynamics of the system if the decision switch is obligately lytic (*P* = 0), stochastic with an equal probability of lysis or lysogeny (*P* = 0.5), or the phage always initiates lysogeny (*P* = 1). In each case, the susceptible cells show a transient increase due to the initial resource concentration, followed by a decline as a consequence of resource scarcity and cellular decay. Lysis also contributes to the decline for the cases of *P* = 0 and *P* = 0.5. In the first case of obligate lysis, the viral population quickly increases and a large number of exposed cells are generated, without the formation of lysogens. In the second case, the viral population does not increase as quickly as in the case of a purely lytic strategy due to the fact that half of the infections generate lysogens (which do not produce more virus particles). The mixed strategy also leads to lysogens (in addition to exposed cells), both of which are considered epidemiological birth states. For the last case of pure lysogeny, there is no increase in the virus particles density given the absence of lysis in the initial decision switch (as noted, the model does not include an induction process over these timescales). This means the virion density rapidly decreases due to decay and infection of host cells. A purely lysogenic strategy does generate both types of birth states, i.e. exposed and lysogens; however, very few bacterial cells are infected. Consequently, the total aggregate density of exposed cells and lysogens is significantly less than that of either the purely lytic or stochastic strategy.

We compare the relative fitness of the purely lytic (*P* = 0), mixed (*P* = 0.5), and purely lysogenic (*P* = 1) cases over a 48-h time horizon. As stated previously, the cell-centric metric of fitness depends on the population of lysogens and exposed cells. Figure 2 compares the birth state population for all three strategies over the time horizon of 12–48 h. Initially, from 12 to 36 h, obligate lysis appears to be the dominant strategy. The use of an initially low ratio of phage to hosts, coupled with the short time horizon, prevents a host population collapse, and a large number of exposed cells are generated through lysis. However, for long timescales, i.e. beyond 36 h, a pure lytic strategy depletes the bacterial population such that the total number of exposed cells begins to decline and the mixed strategy (*P* = 0.5) becomes more favorable. The change in outcomes with timescales implies that phages that adaptively sense and respond to the state of the infection could potentially outperform phages that utilize fixed strategies.

### 3.2 Effect of time horizon on optimal strategies for phage reproduction

We evaluate the near-term phage reproduction of temperate phage using a communication molecule for lysis–lysogeny decisions and show how time horizons shape the responsiveness to the signaling molecule. Hence, rather than using a fixed value of *P*, the infected cells are lysogenized as a function *P*(*A*) where *A* is the concentration of arbitrium in the system. We initialized the system with 10^7^ bacteria and an MOI of 0.01. For each time horizon we use the optimization framework described in 2.3 to calculate the optimal response function to arbitrium molecule—optimal probability function $P_{opt}(A)$—that maximizes near-term phage reproduction.

**Figure 3.**
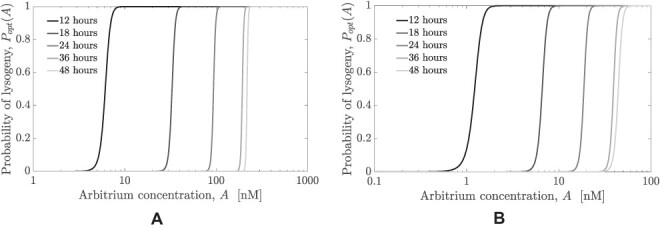
Comparison of the optimal lysis–lysogeny decision response functions (function of arbitrium molecule concentration) given variations in time horizon (from 12 to 48 h). The switching point (from lysis to lysogeny, i.e. from *P* = 0 to *P* = 1) in the sigmoidal lysis–lysogeny response function shifts to the right as the horizon increases. (A) Production rate of arbitrium molecules used is $\mathrm{5\times 10^7 \ molecules/cell\ h^{-1}}$ as given in Table A1. (B) A lower production rate of $ \mathrm{10^7 \ molecules/cell\ h^{-1}}$ is used in this case, which results in a corresponding shift of optimal strategies to lower concentrations.

**Figure A1.**
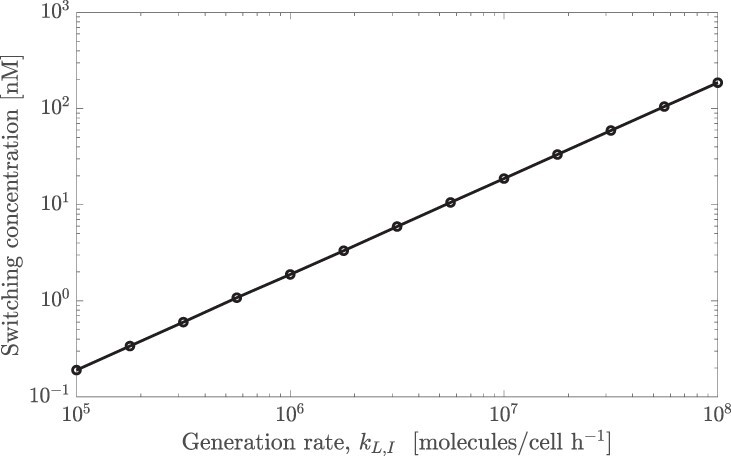
Effect of production rate of arbitrium—$k_{L,I}$, on optimal switching concentration for a time horizon of 24 h. The production rate is varied from 10^5^ to $10^{8}\ \mathrm{molecules/cell\ h^{-1}}$ with exponential steps of 10^0.5^. For each arbitrium production rate, we compute the corresponding optimal switching concentration. The relationship between the optimal switching concentration and arbitrium production rate is linear (correlation coefficient = 1).

Figs. 3A and B show the optimal probability functions for all time horizons between 12 and 48 h, $P_{opt}(A|t_f)$ (*t*_*f*_ being the time horizon for optimization), with arbitrium production rates of 5 × 10^7^molecules/cell h^-1^ and 10^7^ molecules/cell h^-1^, respectively. For the different generation rates, the decision strategy is robust and a corresponding shift in the switching point (from lysis to lysogeny) can be observed. The switching point scales linearly with the production rate of arbitrium (see Fig. A1), maintaining a pattern of an initial lytic period followed by a lysogenic period in the phage dynamics. Therefore, we set the arbitrium production rate to be 5 × 10^7^ molecules/cell h^-1^ for subsequent analyses, recognizing that changes in production rate will also shift the expected switching point as a function of *A*. Likewise, arbitrium responsiveness is nearly identical when the optimization framework maximizes the lysogen production in the near term, highlighting the impact of phage decision switches as a means to maximize the generation of lysogens in the near term as the basis for long-term fitness maximization (Fig. A3). We revisit this point in the Discussion.

For each optimized lysis–lysogeny decision function, the probability of lysogeny is low when the concentration of arbitrium molecule is small and the probability of lysogeny is high when the concentration of arbitrium is high. The switch from lysis to lysogeny is always sharp for every optimal probability function considered here, which indicates that sensitivity to arbitrium is high upon the switch point. The switching points of the optimal probability functions increase with longer time horizons, indicating the phages might decrease their responsiveness to arbitrium for longer time horizons. We note that for time horizons from 12 to 24 h, the probability of lysogeny was lower than 0.5 for $\sim 80$ per cent of the total period, while for a time horizon of 48 h, the probability of lysogeny was lower than 0.5 for $\sim 50$ per cent of the period. The two observations together mean that for longer time horizons, a larger initial period of lysis followed by a switch to lysogeny is optimal. However, the fraction of time for which lysis occurs decreases with increasing time horizon. Another notable trend is that as the time horizon increases, the gap between the switching points decreases, which suggests that the switching points would not increase indefinitely. The observations of the fractional period of lysogeny increasing with the time horizon and reduction in the gaps between the optimal probability functions show that with increases in the time horizon, the optimal strategy places more weight toward generating lysogens, which are longer lived than exposed cells.

The general tendency with respect to the switch point is to generate lysogens when the susceptible host population begins to decay due to the initial period of lysis and magnified when conditions are adverse (as we show in section 3.5). Therefore, increasing the time horizon favors the production of lysogens as a means of absorbing the free phage population produced in the lysis period. We note that the optimal arbitrium concentration for the sharp switch between lysis and lysogeny also depends on the phage efficacy, which itself is modulated by viral traits. The first panels in Figs. A4A, B, and C shows the strong dependence of the optimal switching concentration on the virion decay rate, burst size, and adsorption rate. However, the time at which the switch from lysis to lysogeny occurs is nearly invariant to variations in these parameters (seen in the second panels of Figs. A4A, B, and C), with variations present only for long time horizons. These results demonstrate that the optimal policy is a period of lysis followed by a sharp transition to lysogeny—a feature robust to variation in system parameters.

### 3.3 Population dynamics for optimal switching strategies

**Figure 4.**
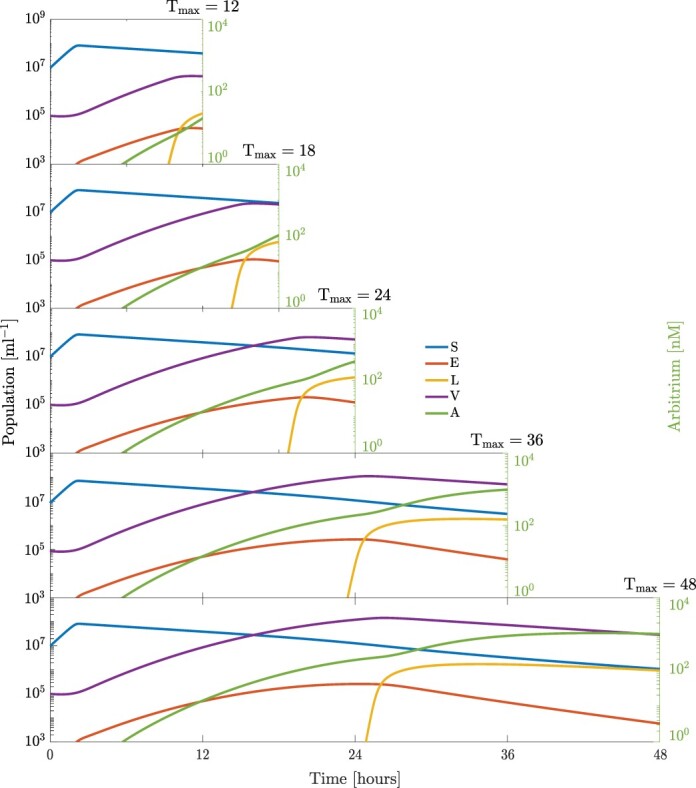
Temperate phage–bacterial infection dynamics for an optimal switching strategy in the lysis–lysogeny decision as a function of the final time horizon. Consecutive panels show an optimal strategy dynamics resulting from the optimal response functions to arbitrium quorum sensing concentration represented in Fig. 3 given a time horizon (12, 18, 24, 36, and 48 h, respectively). We see a common strategy of pure lysis followed by a stochastic strategy in all cases. The time of the switch from obligate lysis to a stochastic strategy can be deduced by the time at which the production of lysogens start. The switch occurs based on the length of the time horizon, with a later switch for longer horizons. The initial MOI is set as 0.01; the additional model parameters are given in Table A1.

We now examine the population dynamics of the full system given optimal switching strategies. Fig. 4 shows the population dynamics for the susceptible and exposed cells, lysogens, and virus particles for time horizons of 12, 18, 24, 36, and 48 h. From the dynamics for all time horizons, we can observe common trends. The susceptible population increases due to the initial resource concentration followed by a decline due to resource depletion, cell decay, and lysis. Next, the concentration of arbitrium molecule takes time to build up in the system; thus, the phages are predominantly lytic at the start. Furthermore, the relative number of phage particles per cell is low; thus, there is an abundance of susceptible cells in the medium. These factors explain the rapid growth of viral particles at the start of the interval and consequently a large population of exposed cells is generated. The end of the initial period of lysis can be observed from the point where the population of lysogens starts increasing. This signals a switch from a predominantly lytic strategy to a lysogenic strategy. In this phase, the viral particle population declines (due to decay and integration with host cells) and lysogens increase. As noted previously, an increase in the time horizon delays the switch to lysogeny. However, the fraction of time with a predominantly lysogenic strategy increases with the time horizon.

### 3.4 Near-term benefits of optimal switching strategies

**Figure 5.**
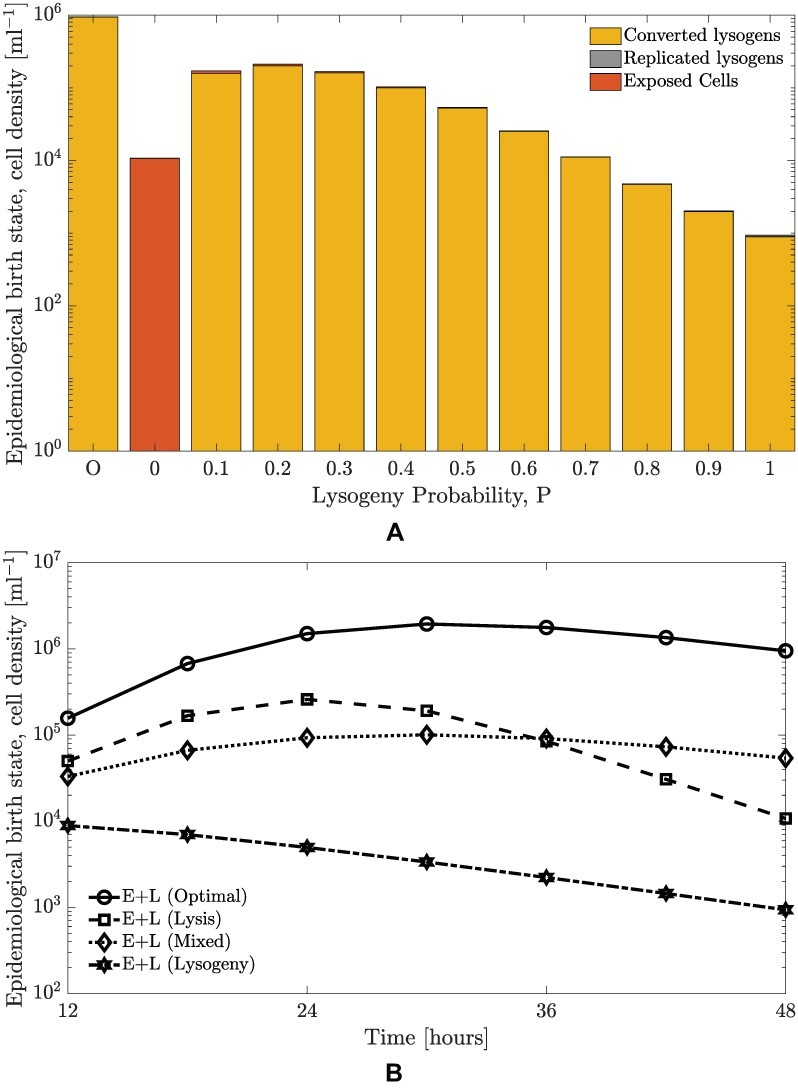
Cell-centric metric of phage reproduction for optimal and fixed probabilities of lysogeny. The relevant parameters are presented in Table A1. Note that the optimal strategy is a response function of arbitrium molecule concentration. The initial MOI is set as 0.01; the additional model parameters are given in Table A1. (A) Comparison between birth states produced from multiple fixed and optimal probability strategies for a time horizon of 48 h with an MOI of 0.01. The cell densities of the converted lysogens, replicated lysogens, and exposed cells are stacked, with the bar representing converted lysogens at the bottom and the bar representing exposed cells at the top. Here, we consider ten alternative fixed probability strategies for lysis–lysogeny i.e. not reliant on arbitrium molecule concentration. We simulate with probabilities from 0 to 1 with increments of 0.1 and compare the performance of all these strategies to the optimal strategy which utilizes the arbitrium system. The comparison shows that the total birth states produced by the optimal strategy is higher than all fixed strategies. (B) The final time birth states population vary with time horizon (from 12 to 48 h) and strategies (optimal, pure lysis, pure lysogeny, and stochastic). The comparison is made for all time horizons from 12 to 48 h, but only three fixed strategies are considered for clarity. The birth states formed by following the optimal strategy are greater than these fixed strategies for all time horizons.

In order to evaluate the benefits of lysis–lysogeny switching based on responses to communication signals, we compare phage reproduction as measured using a cell-centric metric by computing an optimal strategy with that resulting from fixed strategies. Fig. 5 shows the phage reproduction for the optimal probability strategy for 48 h compared against different fixed probability strategies (*P* = 0, *P* = 0.1, …, *P* = 0.9, and *P* = 1). The strategy based on communication performs better than any fixed probability strategy in terms of the sum of the lysogens and exposed cells at the final time. Optimal switching is more than four times better than the highest value achieved for a fixed strategy (for a fixed probability of 0.2), which demonstrates that a phage following such a communication strategy would have a higher near-term reproduction compared to a phage following any other fixed probability strategy. As seen in Fig. A3, the optimal policy is still more than four times better than any fixed policy even if the epidemiological birth states are only lysogens.

Next, we perform the same analysis for all optimal strategies for the time horizons of 12–48 h and compare them to three fixed strategies (*P* = 0, *P* = 0.5, and *P* = 1). Fig. 5 presents the total birth states for all time horizons (12, 18, 24, 30, 36, 42, and 48 h) and compares them to the birth states produced by the fixed strategies. The corresponding optimal strategy produces more birth states compared to any fixed strategy for all time horizons, providing evidence that communication-based switches from lysis to lysogeny may be of adaptive benefit to temperate phage.

**Figure A2.**
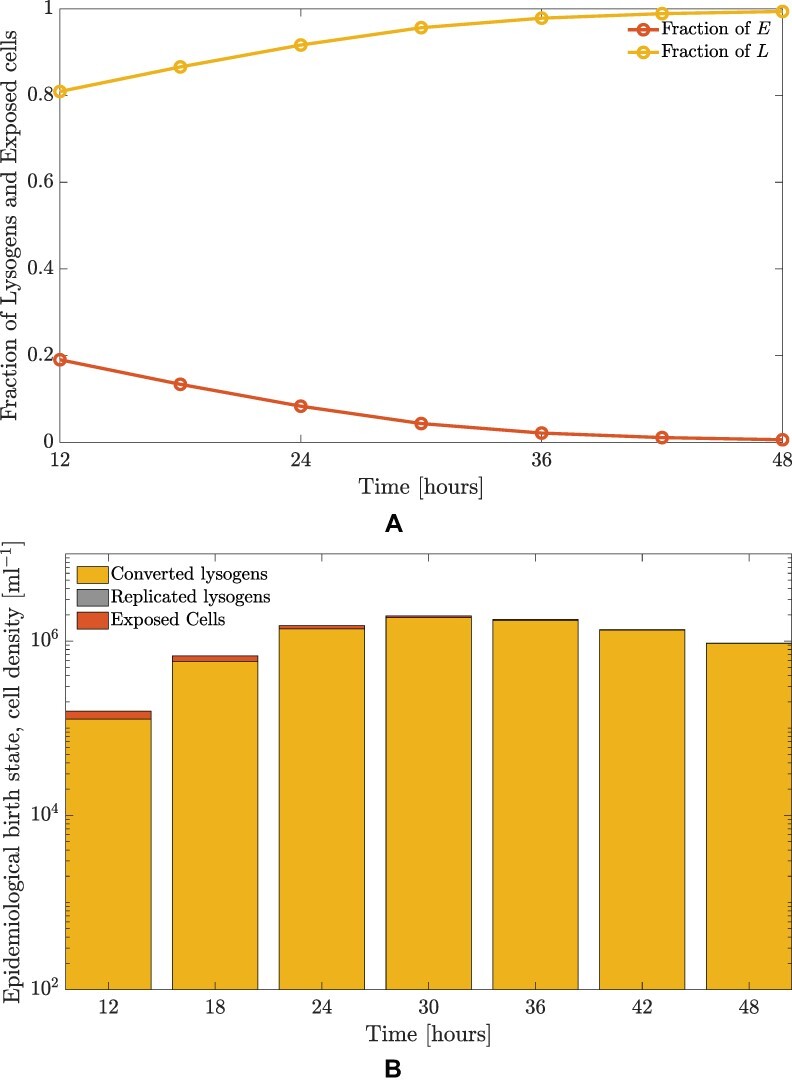
Epidemiological birth state population composition for different time horizons (from 12 to 48 h) when optimal switching strategy is used. (A) Fraction of lysogens and exposed cells at the final time. This shows the relative contribution of the exposed and lysogenic cells to the fitness measure used. (B) Cell density of lysogens and exposed cells at the final time. The cell densities of the converted lysogens, replicated lysogens, and exposed cells are stacked, with the bar representing converted lysogens at the bottom and the bar representing exposed cells at the top. The bar graph also explicitly shows the lysogens created due to conversion and replication. In all cases, the converted lysogens are the dominant part of the birth states.

**Figure A3.**
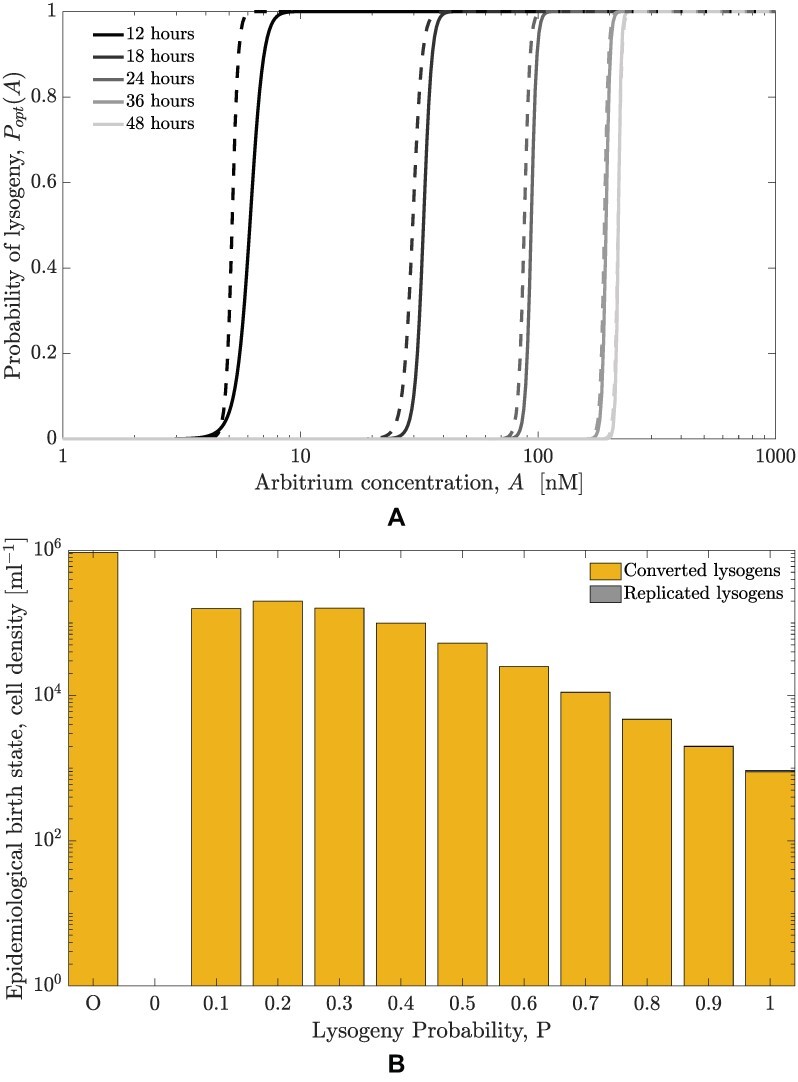
Effect of considering only lysogens as epidemiological birth states. (A) Optimal switching point for time horizons (12 to 48 h) with different objectives. The solid lines represent the optimal probability functions when the objective is maximizing both lysogens and exposed cells at the final time, i.e. $\max E(t_f) + L(t_f)$. The dashed lines represent the optimal functions when the objective is maximizing only the lysogens at the final time, i.e. $\max L(t_f)$. The solid and dashed lines of the same color represent optimal probability functions for the same time horizon. The optimal probability functions are very similar with a slightly faster switch to lysogeny. This increases the lysogen count at the cost of reducing exposed cells, which is immaterial for the new optimization problem. (B) Cell density of epidemiological birth state at final time for $t_f = 48$ h, with the bar representing replicated lysogens stacked on top of the bar for converted lysogens. Similar to Fig. 5, we consider ten alternative fixed probability strategies ($P \in \{0, 0.1, {\ldots}, 1\}$) and compare total birth states. Here, only lysogens are considered as birth states, with the majority being converted lysogens. The optimal strategy is more than four times better than the highest value achieved for a fixed strategy (for *P* = 0.2), demonstrating the higher near-term reproduction compared to a fixed strategy.

**Figure A4.**
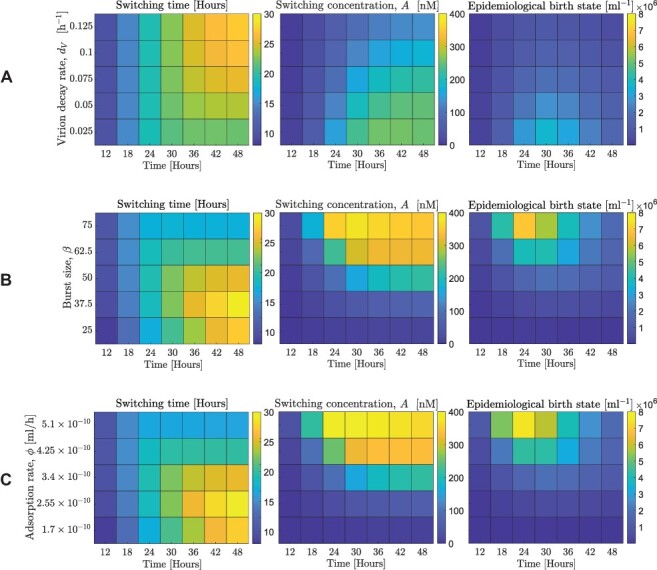
Robustness analysis of optimal switching point for different virion decay rates, *d*_*V*_, burst size, *β*, and adsorption rate, *ϕ*, with the time horizon varying from 12 to 48 h. (A) shows the effect of variation in *d*_*V*_ from 0.025 to $0.125\ h^{-1}$, with $0.075\ h^{-1}$ being the nominal value used in previous plots. (B) shows the effect of variation in *β* from 25 to 75, with the nominal value being 50. (C) shows the effect of variation in *ϕ* from $1.7\times 10^{-10}$ ml/h to $5.1\times 10^{-10}$ ml/h, with the nominal value being $3.4\times 10^{-10}$. In all three subfigures, the first panel (left) shows the actual time at which the switch from lysis to lysogeny occurs, the second panel (middle) shows the switching point in terms of the arbitrium concentration, and the third panel (right) shows the cell density of the epidemiological birth states. The switching time is not robust to changes in the decay rate, burst size, or adsorption rate; however, certain trends remain consistent. Increasing the burst size or the adsorption rate or decreasing the decay rate of virions improves the effectiveness of the viral particles. To prevent host depletion, the switching time is observed to slightly decrease (left panel). Increased effectiveness of the viral particles results in an increase in the epidemiological birth states produced by the final time, as seen in the right panels. The increased population of lysogens increase the concentration of arbitrium in the medium. As the switching time decreases with an increase in virion effectiveness, the switching concentration must increase (due to the increased arbitrium production by the higher lysogen cell density), as seen in the middle panels. While the switching concentration is strongly dependent on these parameters, the switching time changes less due to variations in *d*_*V*_, *β*, and *ϕ*. For variation in decay rates, only slight variation is observed when comparing values for long time horizons. The effect of changes to the burst size and adsorption rate are more pronounced, but limited to long time horizons.

We recognize that measuring total birth states depends on the sum of lysogens and exposed cells, irrespective of composition. To explore shifts in the contribution to fitness, we examined the fraction of lysogens and exposed cells at the final time. As the time horizon increases, the fraction of lysogens monotonically increases from 0.82 at 12 h and approaches 1 at 48 h, with the corresponding reduction in the fraction of exposed cells (see Fig. A2). This implies that given sufficiently long interaction times, optimal switching strategies predominantly rely on lysogens as the basis for increasing near-term fitness. On the cellular level, as stated above, this occurs due to a build-up of arbitrium molecule in the medium over time, leading to inhibition of lysis and switching to a strategy dominated by lysogeny. Further, Fig. 5 demonstrates that the major contribution to epidemiological birth states comes from ‘converted lysogens’, that is, lysogens generated due to infection. However, in order to increase lysogens at the final time, the strategy initially tends toward lytic outcomes. Due to the optimal switching strategy initially favoring lysis, the lysogens start getting produced when the resources have already been consumed, which prevents lysogen replication. Lysis depletes susceptible cells, which reduces niche competition and decreases the potential benefits of horizontal transmission.

### 3.5 Effect of resource level on optimal strategy

Thus far we have evaluated changes in adaptive switching strategies on near-term phage reproduction given an environment without influx of resources for cellular growth, analogous to batch culture conditions. Next, we evaluate the impact on different resource conditions, including (1) increased initial concentration of resources and (2) resource influx which maintains higher levels of resources in the environment throughout the focal period similar to chemostat growth conditions. As before, we use the optimization approach described in 2.3 to identify an optimal strategy, including an optimal switching point, *A*_*c*_ where the system tends to switch from lysis to lysogeny.

**Figure 6.**
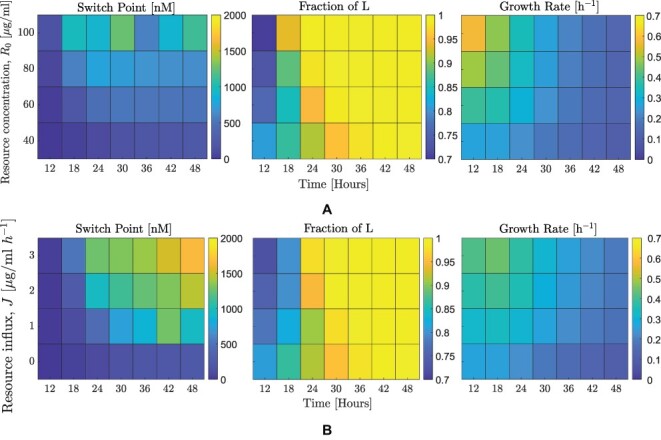
Effect of resource level (change in initial concentration and influx) on optimal lysis–lysogeny switching point. The simulation parameters are indicated in Table A1. (A) Heatmaps for time horizons from 12 to 48 h and variation in the initial resource concentration, from 40 $\mu g/ml$ (which is the base case concentration used in all previous plots) to 100 $\mu g/ml$. (B) Heatmaps for time horizons from 12 to 48 h and variation in resource influx, from 0 $\mu g/ml\ h^{-1}$ (which is the base case concentration used in all previous plots) to 3 $\mu g/ml\ h^{-1}$. In both subfigures, the three panels correspond to the optimal switching point (left panel), fraction of lysogens to infected cells at the final time (middle panel), and growth rate (right panel).

**Figure A5.**
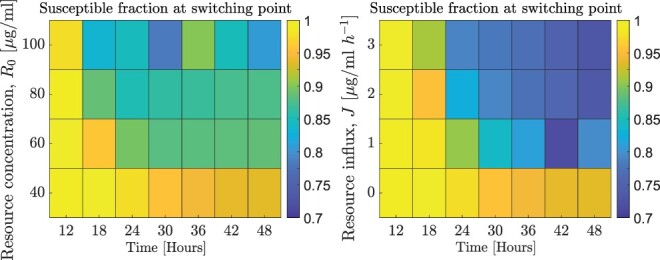
Effect of resource level (change in initial concentration and influx) on fraction of susceptible cells at the optimal switching point. The simulation parameters are present in Table A1. The first panel corresponds to a heatmap for time horizons from 12 to 48 h and variation in initial resource concentration, from 40 $\mu g/ml$ (which is the base case concentration used in all previous plots) to 100 $\mu g/ml$. The second panel is a heatmap for time horizons from 12 to 48 h and variation in resource influx, from 0 $\mu g/ml\ h^{-1}$ (which is the base case concentration used in all previous plots) to 3 $\mu g/ml\ h^{-1}$. An increase in resource concentration in the system (either through increase in initial resource concentration or resource influx) leads to a lower susceptible fraction at the switching point. This translates to a longer period of lysis (which would reduce the population fraction of susceptible cells in the system) followed by a switch to lysogeny. A longer time horizon results in a lower susceptible fraction at the switching point. Both observations are consistent with previous results, where a higher resource concentration or time horizon meant an increase in the arbitrium switching concentration, that is, a longer period of lysis.

Fig. 6 shows three heatmap plots of the optimal strategy for different time horizons and initial resource concentrations. In all three panels the time horizon varies from 12 to 48 h, and the initial resource concentration starts from a base value of 40 $\mu g/ml$ up to 100 $\mu g/ml$. The left panel shows the optimal switching point for a given time horizon and initial resource concentration. For any particular time horizon, as the initial resource level increases, the switching point also increases implying a longer period of a lytic-like strategy. This delay in switching occurs due to the susceptible population rising to a higher value (due to the greater initial resource concentration). However, we also note that the switching point need not inevitably increase for longer time horizons, *t*_*f*_ (see cases of initial resource concentration of 80 and 100 $\mu g/ml$ in Fig. 6). This discrepancy can be explained as follows. For these two initial concentrations, the susceptible population rapidly increases due to the abundance of resources. Given sufficiently short time horizons, phages can maximize fitness by preferentially producing more lytically infected cells, which imply a higher switching point. This strategy of investing in lytically infected cells works in the limit of higher resource concentration, which prevents a population collapse for shorter periods. For longer periods, we again see a return to a lower switching point, which would translate to investing in the production of lysogens. The fraction of lysogens (middle panel) increases with an increase in time horizon, as noted in the previous section. This trend is true for all initial resource concentration values. We observe a monotonical increase in the growth rate of phage with the initial resource level (right panel, Fig. 6). However, as time horizon increases, the growth rate is observed to decrease. The reduction suggests that environmental factors that alter host populations, such as nutrient depletion, may drive selection of communicating phage to become more sensitive to arbitrium concentration when conditions are less favorable for the host. The initiation of lysogeny when hosts are scarce increases the chances of increasing infected cells, albeit in an integrated form. However, when we look at the fraction of susceptible hosts in which the switch to lysogeny occurs it varies with the resource level (Fig. A5), indicating that the optimal arbitrium concentration does not depend on the population composition but on the overall dynamics to the final time.

Figure 6 shows three heatmap plots of the optimal strategy for different time horizons and resource influx. In all three panels the time horizon varies from 12 to 48 h, and the resource influx varies from 0 (no influx) to 3 $\mu g/ml \ h^{-1}$. The left panel shows the optimal switching point for a given time horizon and resource influx. For any particular time horizon, increasing the resource influx also increases the switching point, as before. Further, the switching point concentration of arbitrium molecule strictly increases with increasing time horizon for the considered values of resource influx. The susceptible population does not increase as rapidly, which we interpret to mean that the optimal strategy is for phage to invest in lysogens. The fraction of lysogens (middle panel) also follows a similar increasing trend with an increase in time horizon and is true for all resource influx values. Finally, the right panel shows the growth rate. Again, a higher influx translates to a higher growth rate. Similar to the trend observed before, the growth rate decreases with an increase in time horizon.

## 4. Discussion

We have developed an optimal control framework based on the SPbeta phage arbitrium system to study the near-term adaptive benefits of responsive switching between lysis and lysogeny. We used a cell-centric metric to compare temperate phage reproduction in the near term. Optimal strategies computed through this technique all share the common feature of exhibiting a rapid switch from high probability of lysis when the concentration of arbitrium molecules are low to a high probability of lysogeny when the concentration of arbitrium molecules are high. The longer the horizon, the later the switch occurs, allowing for an increase in infected cells. Thus, for temperate phage, an initial period of lysis allows sufficient growth to infect a large number of bacterial cells. After this, a switch to lysogeny ensures that the host is not depleted and the phage generates a large number of infected cells in the form of lysogens. Initial resource concentration or influx, creating a higher resource concentration, results in comparatively longer periods of lysis followed by lysogeny. This exploits the increase in bacterial population due to higher resource concentration which can support a larger viral population without collapsing. The switching point is expected to increase in systems with more resources and/or those that can support sustained periods of host growth.

In exploring optimal adaptive strategies, we find that the time horizon plays a role in another emergent property: the relevance of lysogeny. For longer time horizons, the fraction of lysogens present at the final time increases. This occurs due to a buildup of arbitrium molecules in the medium, which increases the probability that infected cells initiate the lysogenic pathway. In this framework, we interpret the preferential use of lysis as a short-term strategy for immediate increase in phage particles and lysogeny as part of a longer time strategy to maximize the growth of the infected cell population (which we use as a metric for near-term phage reproduction). Although the growth rate declines for long time horizons, lysogeny ensures that the phage can persist despite a depleted host population, which is not possible if lysis is continued indefinitely. We note that the inclusion of near-term time horizons independent of intrinsic dynamic timescales has been shown to lead to important constraints enabling transitions from solitary cells to multicellular groups (Black, Bourrat and Rainey 2020).

Even with this simplified model, we observe interesting properties which can shed light on the balance between the lysis and lysogeny pathways. In our model, phage that utilized a fixed strategy of lysis, lysogeny, or stochastic (but non-responsive) switching had lower reproduction rates than phage with optimally responsive switching strategies. Thus, there is a near-term advantage conferred by temperate phage that sense and ‘choose’ the optimal life cycle (lytic or lysogenic) (consistent with the findings of Doekes, Mulder and Hermsen (2021), albeit here we provide evidence in support of sharp switching, rather than assuming such switching is sharp a priori). Notably, these findings hold for resource-limited conditions, but increases in resource availability can shift the optimal strategy toward lytic outcomes (especially in the short term). Connecting phage strategies with environmental conditions will require considering strategies that include a continuum of potential outcomes whose benefits may change over time and environmental context (Correa *et al.* 2021).

The optimization approach used here has certain limitations. The optimal control method identifies the switching strategy given a fixed time horizon. However, there is no guarantee that this strategy is an evolutionarily stable strategy over the long term. In related work, Doekes et al. used an eco-evolutionary modeling framework to explore the potential evolution of fixed decision strategies (Doekes, Mulder and Hermsen 2021). Systematic examination of how viral genomes pass from one environment to the next may help bridge the gap between our short-term analysis and evaluation of long-term strategies. In doing so, the full life cycle will need to be included (e.g. see Lion and Gandon (2022) for relevant, related analysis). For example, spontaneous induction has a negligible effect on the adaptive benefits of switching in the near term. However, the induction of prophage leading to the reinitiation of the lytic pathway would become especially relevant in the long term. Recent work shows that communicating phage restarts lysis via arbitrium induction, preventing spontaneous induction via DNA damage (Aframian 2022) and triggering prophage induction when signal concentrations decline, potentially indicating the presence of new susceptible hosts nearby (Bruce *et al.* 2021). Moreover, if the initial MOI is very low or the environment is resource-rich, then a pure lytic strategy (rather than a switching strategy) could have higher fitness for short-term horizons. Finally, the cellular sensing mechanism for arbitrium molecules is a complex process which was simplified here by assuming the sensed concentration follows a Poisson distribution based on the mean concentration of arbitrium molecule in the medium—extensions should include the intracellular uptake and regulation of decisions based on internal arbitrium ‘quotas’. Nonetheless, our finding that sharp transitions are optimal in the near-term provides additional context for experimental and modeling studies of the long-term evolution of arbitrium-based switches. The integration of a changing ecology and a sensing mechanism based on cellular mechanism would improve the applicability of the model in predicting optimal strategies for phage in real environments. In doing so, it will be critical to investigate the dependence of the optimal strategy on the multiplicity of infection.

In summary, the control-theoretic framework developed here demonstrates additional support for the adaptive value of a sensing mechanism for initiating lysis or lysogeny based on environmental context informed by prior infections. In doing so, our results suggest that the early lysis of cells by temperate phage provides a mechanism for rapid expansion of virus particles that can then, later, preferentially initiate lysogeny given sensing of an extracellularly released communication peptide. When viewing the reproduction of viruses in terms of infected cells, such changes in strategies represent a form of feedback control by which phages both modulate and reshape their ecological context. Moving forward we hope that this near-term control-theoretic framework is useful in evaluating how such sensing mechanisms may evolve over longer time horizons.

## Appendix

**Table A1.** Parameter choices from previous works (Weitz 2015). $\dagger$, $\ddagger: k_{L,I}$ estimation from data in (Erez *et al.* 2017) (see 2).

Parameter	Value	Unit
*e*, conversion efficiency	$5\times 10^{-7}$	$\mu g$
$\mu_{max},$ maximum growth rate of cells	1.2	*h* ^−1^
*R*_*in*_, half saturation constant	4	$\mu g/ml$
*λ*, transition rate from exposed cells	2	*h* ^−1^
*η*, lysis rate	1	*h* ^−1^
*J*, influx rate of resources	0	$\mu g/ml h^{-1}$
*d*_*R*_, decay rate of resources	0	*h* ^−1^
*d*_*S*_, cellular death rate of susceptible cells	0.075	*h* ^−1^
*d*_*E*_, cellular death rate of exposed cells	0.075	*h* ^−1^
*d*_*L*_, cellular death rate of lysogens	0.075	*h* ^−1^
*d*_*I*_, cellular death rate of lytic cells	0.075	*h* ^−1^
*β*, burst size	50	
*ϕ*, adsorption rate	$3.4 \times 10^{-10}$	$ml/h$
*d*_*V*_, virion decay rate	0.075	*h* ^−1^
*α*_*S*_, selection coefficient	0.1	
*k*_*L*_, generation rate of arbitrium molecules from lysogens	$5\times 10^{7 \dagger}$	$\mathrm{molecules/cell\ h^{-1}}$
*k*_*I*_, generation rate of arbitrium molecules from lytic cells	$5\times 10^{7 \ddagger}$	$\mathrm{molecules/cell\ h^{-1}}$
*d*_*A*_, decay rate of arbitrium molecules	0.1	*h* ^−1^
